# Altered eating: a definition and framework for assessment and intervention

**DOI:** 10.1186/s40795-018-0221-3

**Published:** 2018-03-27

**Authors:** D. L. Burges Watson, S. Lewis, V. Bryant, J. Patterson, C. Kelly, R. Edwards-Stuart, M. J. Murtagh, V. Deary

**Affiliations:** 1 Institute of Health and Society, Newcastle University, Newcastle Upon Tyne, UK; 2 Department of Geography, Durham University, Durham, UK; 3 Durham, UK; 4 City Hospitals Sunderland NHS Foundation Trust, Sunderland, UK; 5 Freeman Hospital Northern Centre for Cancer Care, Newcastle Upon Tyne Hospitals NHS Foundation Trust, Newcastle Upon Tyne, UK; 6 London, UK; 7 Newcastle University, Newcastle Upon Tyne, UK; 8 School of Life Sciences, Northumbria University, Newcastle Upon Tyne, UK

## Abstract

**Background:**

Eating can be a significant challenge for cancer survivors; however, to date there is no systematic way of assessing and addressing food related quality of life in this group. The purpose of our study was to develop a framework for doing so.

**Methods:**

Over the course of 6 years in participant-led food workshops, we worked alongside 25 head and neck cancer (HNC) survivors and their partners, employing video-reflexive ethnographic (VRE) methods. The current study reports on data from the two summative workshops of this series where we worked with participants to cohere the emergent themes. Video and transcripts were reviewed and coded with participants and stakeholders according to domains of life that were affected by food. Three of the authors, one of whom is both survivor and researcher, arrived at the consensus framework.

**Results:**

Seven areas of life were identified as affecting, or being affected by, altered eating. Three were physiological: *anatomical, functional* and *sensory*. Two captured the *cognitive* and *behavioural* labour of eating. *Social life and identity* were altered. The foregoing had an enduring *emotional impact.*

**Conclusions:**

Altered eating has physical, emotional and social consequences. The altered eating framework provides a systematic way of exploring those consequences with individual survivors. This framework has the potential to improve both the assessment and treatment of altered eating, to benefit food-related quality of life.

## Background

Eating can pose profound challenges for patients recovering from cancers including head and neck, oesophageal, lung and bowel. In advanced cancer it is estimated that more than 50% of survivors experience concerns about weight loss and loss of appetite [1]. A recent systematic review of eating difficulties in cancer emphasises that there are no effective interventions for improving food-related quality of life, no consistent assessment methodologies, confusion in terminology regarding sensory issues, and little account of the importance of food hedonics and other aspects of what makes food enjoyable to eat [2]. Most research has looked at eating-related difficulties during treatment, with evidence that chemosensory changes may resolve around 8 weeks after treatment ceases. An emergent body of qualitative research has identified how treatment and cancer can have long term impacts on the ability to ‘eat well’ long after treatments ceases [3–12]. Ganzer et al’s (2015) review of qualitative literature found several consistent themes across studies that demonstrate a ‘significant impact’ on the experience of eating and changed meaning of food. The themes cross social, psychological and cultural issues in addition to the more commonly reported physiological and functional difficulties [11]. Ganzer et al conclude that further studies should consider ‘not only the functional impact associated with treatment for HNC but also the social and emotional context of eating’ [7]. Their conclusion also follows the systematic review of Cousins et al [13] in identifying the tendency for researchers to focus on the (often short term) medical complications of treatment with (chemo)radiotherapy and the impact of dysphagia (swallow difficulties) [14]. Authors stress the necessity to look beyond functional difficulties to consider a more ‘holistic’ approach [6] that also addresses emotional and psychosocial issues and support needs [13, 15].

A more ‘patient centred’ and less ‘disease-focused’ approach has been advocated as a means to address these broader concerns [15]. Patient and public involvement (PPI) in research is increasingly valued in health research in which research is done ‘with’ rather than to patients [16–18]. In previous work we have identified how co-production can be more meaningful and patient/survivor-focused in its questions, research processes, planned milestones, outcomes and methods of dissemination [19]. Qualitative research methods are of value in drawing out phenomenological experience; to our knowledge, no study has employed a co-productive approach to explore eating difficulties in head and neck cancer survivorship.

Losing the ability to eat well, what we call ‘altered eating’, can reduce quality of life and have serious consequences for physical and emotional health and wellbeing [20–22]. In the course of illness and treatment, clinicians’ focus is necessarily on the condition underlying or causing altered eating, alongside managing its nutritional consequences and improving eating-related function where possible. However, as our study shows, altered eating can persist after treatment or become an enduring feature of patients’ lives.

Despite the prevalence of altered eating, there are no comprehensive guidelines or pathways to address explicitly the impact of changes in the experience of food and eating in public health, general practice or other clinical settings. In our work with head and neck cancer survivors, it became clear that to fully assess and address issues related to food quality of life, a comprehensive framework that addresses and builds on thematic areas identified in qualitative work, was necessary. Head and neck cancer survivors offer an excellent exemplar of altered eating for the systematic development of such a framework, in that they encounter some of the most complex combinations of physiological, emotional and psycho-social eating difficulties of any patient group [7]. In our work with this group we sought to derive a multi-dimensional framework from co-produced, comprehensive accounts of survivors’ altered relationship with food, which would facilitate systematic assessment of these issues and thus form the basis for a more comprehensive intervention.

## Methods

This framework evolved over the course of 6 years’ collaborative work with: head and neck cancer survivors: chefs and flavour experts (SS, RES): a Speech and Language clinician (JP): an oncologist (CK); inter-disciplinary social scientists with expertise in sensory ethnographic methods (DBW, SL) and health psychology (VD). Video-reflexive ethnography (VRE) is a collaborative methodology involving the negotiated videoing of everyday practice around health and healthcare, and a reflexive process in which participants help make sense of the video footage they have helped produce or feature in [23, 24]. Given the centrality of survivors’ experience of food and eating, we adopted a multi-sensory approach that paid attention to the embodied experience of food ‘beyond talk’ [25]; that is, beyond the interview and focus group research that has tended to dominate the field. Multi-sensory ethnographic methods attend to forms of intimacy, sociality and emplacement and to the complete range of sensory experience including visual, sound, taste, smell and touch [26, 27].

We instantiated these principles in four pilot “food play” workshops and a further 16 food and eating workshops undertaken as part of a research project entitled *Resources for Living (R4L) Pilot: Exploring the Potential of Progressive Cuisine for Quality of Life Improvement for Head and Neck Cancer Survivors* [NIHR number PB-PG-0711-25,040]. Potential participants were approached through post-treatment head and neck cancer survivor support groups, by Speech and Language Therapists from two services in the North East of England. Participants were excluded if they were still receiving treatment or wholly dependent on Percutaneous Endoscopic Gastrostomy (PEG) tube feed or Sip feeds (oral nutritional supplements). Inclusion criteria were as follows: post-treatment, in a relatively stable state of health and able to eat by mouth.

Food play workshops involved a chef skilled in techniques of modernist or ‘progressive’ cuisine employing insights from sensory science to improve flavour, textures and the hedonic qualities of food. Each week a new food group (e.g. “Smoothies”, “Stocks”, “Chocolate Mousse”) was explored, usually at the prior request of participants; at the end of each workshop we revisited discussions about what foods participants missed and what the chef had in mind, and consensus was reached on the focus of the next workshop. These groups involved lively discussion, play and experimentation with food.

In the following derivation of the framework, we exemplify key framework components from participant reflections in two summative sessions, conducted towards the end of the workshop series. Here, we asked participants to reflect on the way their relationship to food had changed because of their illness, and how this had affected their and their families’ lives. From the co-produced analysis of data from previous workshops we had realised that *loss of pleasure* in food, and the *increased effort and burden* of food were central issues for this group, so pleasure and burden were additional focus prompts for the two respective groups. However, these only served as starting points for discussion, and participants were encouraged to elaborate on the causes and consequences of altered eating in their lives in general. As a result, participants reiterated and expanded upon discussions that had occurred in the previous workshops, but also brought out new examples and reflections.

### Analysis

Food play workshop video data was analysed iteratively (by DBW, VB and SL), following each session in the series of food play workshops. A template, based on our PPI work, was used to record the findings of each workshop (e.g., participants’ food memories, coping tips, foods most missed, verbal and observed taste and flavour responses), ensuring that anticipated and emergent themes and participant feedback were recorded for comparison. The template used to record the workshops is available from the corresponding author on request. Themes were then explored in more depth in summative sessions with those participants able to attend. To derive a comprehensive framework, data from those summative sessions was then coded according to domains of life that were affected with regard to food. This produced a range of three broad categories: biological causes; psychological consequences and social impact. A second round of coding sought to further discriminate areas within these broad fields that were meaningfully distinct from each other. Two of the authors (DBW and VD) did this coding separately and then with VB, who is both survivor and researcher, arrived at a framework comprising seven areas within the previous three broad categories. Finally, participants and stakeholders worked collaboratively to produce video material that exemplified their experience of altered eating using these framework components as a structuring device. The resulting short film [28] was then presented to survivors, carers and clinicians at two public film screening events (*N* = 80) with opportunities for all participants to offer feedback and comments. The agreed final framework comprised three domains containing seven features: 1. patient physiology (anatomical, functional and sensory); 2. patient labour (behavioural and cognitive); and 3. patient identity (cultural/social and emotional).

## Results

**Fig. 1:**
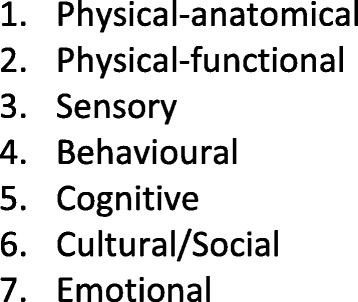
Altered eating framework

In the following we present the framework (Fig. 1) as it emerged from the thematic findings and follow up stakeholder events. We provide representative quotes from survivor participants to exemplify each area. To put these in context we have given the reader some essential references to explain issues such as impacts on the swallowing mechanism, and key dimensions of flavour perception. The first three elements – the anatomical, functional and sensory changes – are fairly well established in the literature, and most of the research tends to focus on these aspects. There is, however, less emphasis on the phenomenology (as opposed to the physiology) of altered functional and sensory experience, and very little on the behavioural, cognitive, social and emotional elements.

### Participants

Over the course of the study a total of 25 participants and their partners were recruited. The participant survivors had all received (chemo)radiotherapy as treatment and were 6–60 months post-treatment but with on-going difficulties with food and eating. The majority (*n* = 14) were males between the ages of 54–65. Participants came from a diversity of socio-economic backgrounds but with similar cultural links to the North East of England. Over the course of the workshops a number of participants (*N* = 5) were lost to the study due to ill-health or death. Not all participants were available to attend the final two summative workshops. Present at both summative sessions were survivors (*N* = 10 Group 1 R1-R5, Group 2 R1b-R5b) and partners (*N* = 4), two social scientists, one health psychologist, one chef and a documentarian who recorded the sessions. One of the head and neck cancer survivor participants was also part of the research team and had received training in qualitative methodology.

#### Anatomical: The anatomical structures required for eating are altered.

All of our participants had changes to the throat, upper oesophagus, salivary glands, mouth, tongue, teeth and/or other structures, which impacted on the movement of food from the mouth to stomach, and/or increased the risk of choking.

R3b: They did one of those video swallow tests and it materialised that there was like a web growing across my gullet, so that was the reason why it was becoming more difficult, so they did a gullet stretch, which initially was good for maybe about a week and then it went back, then I had another gullet stretch and at that one I had real breathing difficulties, so because of that he doesn’t want to do anymore gullet stretches and gradually it’s become more and more difficult to swallow anything.

These changes are often a result of the treatment rather than the cancer itself:

R1b: They were going to basically fry my saliva glands on the right side.

From the literature it is known that the severity of swallowing problem will depend upon the extent of the disease, the structures involved and the type of treatment [29]. For example, surgery for a tongue cancer will involve removing important swallowing structures, leaving additional spaces and sumps (holes where liquid/food collects) within the mouth:

R2b: With me it’s under my tongue, because they took a slice off my tongue and I’ve got that cavity where it [food] can sit and I can’t move that part of my tongue.

Almost all head and neck cancer patients treated with radiotherapy experience mucositis of the mouth and throat, which can make eating and drinking extremely painful [30]. Damage to the salivary glands results in xerostomia, or dry mouth [31].

R3b Whatever I eat has to be accompanied by lots of liquid, which also dilutes the taste of the food, which is another thing.

In the long term, xerostomia increases the likelihood of dental caries and tooth extraction, impacting on the ability to chew and, potentially, in tooth loss:

R4: Mine snapped off when I was trying to eat.

Post-treatment oedema can reduce the natural ‘drip trays’ of the swallowing mechanism, increasing residue and the potential for aspiration making the act of swallowing difficult [32]:

R3b: Gradually it’s become more and more difficult to swallow anything. I do eat what I can, but it’s a huge effort, it takes ages.

#### Functional: The act of eating is altered

Swallowing is a highly co-ordinated, continuous and complex sequence of motor and sensory behaviour. Both cancer and its treatment can significantly alter swallowing physiology [33, 34]. Besides these purely anatomical impediments reported above, participants also experienced a change in the co-ordinated act of swallowing. Pre-treatment this is was an automatic behaviour, but now it often required conscious control. In addition, it had acquired the characteristics of a risky and unpredictable behaviour, one which would sometimes work, and sometimes go wrong:

R1b: I’m reminded of the response like when you’re a child and we used to go to the river and jump off a really high bank and there were different high banks and you’d go, one, two, three, go, but your feet wouldn’t move and your knees wouldn’t let you and your head was saying jump, but your body was saying, no way! …. That’s what it’s like when I get that bit of meat in the back of [my] throat.

Participants acknowledged that beyond anatomical alterations, previous experience of choking made them tentative about moving the food bolus from the mouth towards the oesophagus:

R1b: It’s my brain saying, don’t, because you’re going to end up, up the creek without a paddle, blue lipped and I think it’s just my body’s mechanism saying, no, don’t, that’s it, that’s over.

This caution and fear may be increased by surgery and radiotherapy effects, both of which can lead to sensory deficits, reducing proprioception of food and liquid and potentially inhibiting the protective cough reflex, should the bolus fall into the airway [35]. Overall the swallowing process – something that once could be initiated and sustained fairly automatically – has become something the smooth functioning of which could no longer be relied on; one that required management:

R1b: Yeah, see and what I’ve also found after with the treatment was my swallowing, the actual mechanism got very clunky at times.

#### Sensory: Flavour perception is altered

Flavour should be distinguished from taste. Where the latter comprises the sensory inputs of sweet, sour, bitter, salty and umami, flavour is currently understood as the totality of sensory experience in relation to food and eating, including taste. Multi-modal flavour perception involves a fusion of taste, trigeminal nerve stimulation, sounds, visual cues, temperature, texture and smell that are unified in the act of eating and savouring [36, 37]. This distinction is important as for participants it was not just taste that was altered, but the sensory gestalt (or totality) of flavour. As one participant observed:

R1: There’s a load of triggers for you to enjoy your meal. You go for a meal, right, you go into a restaurant, and first you walk through the door you can smell it, you go and sit down, you look through a menu and you start ordering and it looks lovely, then they bring it to the table and you actually visualise, you see it, that meal and that looks, oh fab, and then you start, it all starts the whole, what’s the, process of having a meal. It’s all and I think you need all of them triggers for you to enjoy your meal properly

Thus when participants talked of the loss of taste, such as “I’ve got very little taste for anything” (R3), it may be implied that it is not just taste but the overall perception of food flavour that is altered. Elements such as the texture, temperature and ‘spiciness’ of food, mediated in part by the trigeminal and olfactory nerves [38], may become intolerable, for example:

R1b: I remember the first time I tried chocolate, it was like sticking a spoonful of axle grease in my mouth honestly, oh, it’s the worst thing I’ve ever experienced.

R2: Curry was the one thing I missed and was unable to eat at all, because of the spices and the pain they caused in my mouth.

The length of time taken to eat leads to food becoming cold, again altering the experience of flavour:

R4: You can start a meal, you can have a couple of mouthfuls, it goes cold, you don’t want it.

It was also the variability and unpredictability of flavour perception that was reported as frustrating, making eating hard to manage:

R1: Yeah, like I say, sometimes you can have a meal and then a couple of weeks later you go back to the same meal and think, yeah I’m going to enjoy this, and it’s eurgh, it’s totally different and you think, what’s going on like. It’s confusing sometimes.

R5: My taste changes day to day, hour to hour. Sometimes I can taste something, sometimes I can’t.

R1: Sometimes I take a forkful of curry and it’s just, oh, it’s just a bliss sensation, then the second forkful nothing, absolutely nothing.

The research literature suggests that the loss of saliva as a result of xerostomia may affect taste and smell perception, since saliva is used to carry taste compounds to the taste receptors on the tongue via chewing and swallowing [39, 40]. Saliva also performs a protective function [41]; its absence can increase sensitivity to spicy or acidic foods:

R5: Whatever you buy, like you buy mince one day, a different mince and it makes your tongue burn and then that, when you try a glass of wine with that, that’s even more burning.

Loss of taste and taste dysfunction are strongly associated with loss of flavour perception and therefore enjoyment and satisfaction [21] leading to diminished appetite, and consequent weight loss [42]. As we shall see later (emotional impact) some participants had lost interest in food altogether as a direct result of changes in flavour perception.

#### Behavioural: The routines of food preparation and eating behaviour are altered.

Participants reported that their food related behaviour had altered substantially, with regard to planning and preparation, the act of eating, and in terms of “acts of recovery” required to overcome the effects of eating.

In terms of eating, participants frequently described it in terms of *effort*:

R1b: it’s a chore basically, eating now, it’s a real chore.

Part of what had turned food into a chore was the duration of the activity; eating now took much longer:

R5: My other thing is that I have to eat slowly. It takes … I mean everyone else has finished and I’m still … twice as long.

R1: Which is a big thing and all because once your food starts to go cold it’s even harder to get it down. I’m going to have to tube feed, yeah, I’m just getting fed up with trying to eat, it takes so long, it’s just a chore,

The efforts required to find and prepare something to eat also took up more time:

R1b: I spend hours traipsing round up and down every aisle in the supermarket looking, could I do that, could I eat that, I don’t know, give it a try. You get it home, try it, prep it…

As did the acts required to recover from eating:

R5; And one of the worst things is getting rid of the detritus or whatever you call it. That going out to eat and you have to find somewhere to put the tooth things and clean your throat and that is really socially off-putting.

R1: I do my teeth at least four times a day, at least four times a day, because I don’t want to lose them. They’re in a right state as they are, but it’s only over the last four and a half years that they’ve got like that.

Overall for this group of head and neck cancer survivors, post-treatment eating entailed smaller quantities of food being chewed for longer, associated with increased effort and consequent fatigue. As one participant remarked, eating had become “tiresome” (R3b). This helps make sense of the research that shows that patients intent on weight gain make calculations of anticipated effort of ingestion versus calories ingested and opt for higher calorie/low effort food [3]. As such, ease rather than enjoyment is the criterion often shaping food choices.

#### Cognitive: The amount and type of thought concerned with food is altered

The increased behavioural labour and concomitant exhaustion was matched by the increased cognitive effort that food entailed, often at the expense of other tasks.

R1: You’re so conscious of it [eating], you’ve got to concentrate so much.

Psychologists distinguish between two kinds of cognitive processes involved in the initiation and maintenance of behaviour [22]: automatic, implicit, processes governing rote and habitual behaviours and reflective processes governing complex cognitively demanding behaviours. Eating is normally an automatic process, or it certainly can be. However, for this group, failure to pay attention to the texture of food within the mouth, and to deliberately manage the act of swallowing, could result in choking and/or aspiration. This resulted in an enforced experience of “mindful eating”; i.e., an intense explicit reflection on the processes of chewing and swallowing. This made eating an insular experience, even in company:

R2: I can’t join in the conversation with anybody else … because I can’t control the food in my mouth and I need to concentrate so hard on what I’m doing, you’re not part of the social group anyway

As eating required increased cognitive attention, so did the planning and preparation of food:

R1b: You do have to put a lot of thought into it and how you’re going to cook it and how much you cook it.

Participants who were still engaged with trying to manage their eating described detailed planning of meals, with each meal being an experiment that required attention and careful monitoring; an experiment that could go wrong:

R1: It looks beautiful and then when you sit down you’ve got to start thinking, how am I going to eat this, how am I going to do this, am I going to have problems. You worry. I worry about what’s going to happen.

#### Social and cultural: Social participation and social identity are altered

Commensality, or eating together, especially at the same table, plays a fundamental role in creating and reinforcing social relationships [43]. Participants reported alterations in this dimension of eating too:

R1: I won’t go out for a meal now, because I’ve stopped doing that because I had a session in Frankie and Benny’s where I started choking and people just step over you.

R2b: Half the time you don’t want to go out because you feel self-conscious that people are watching to see what you’re up to.

Given the cognitive labour entailed by eating, described above, for many eating had lost its social dimension entirely and become a purely private experience:

R2: The only time I really enjoy a meal now - a meal, a small amount of food - is when I’m on my own and that is the only time.

Losing the ability to eat in public, or even with friends and family, risks destabilising the social foundations on which human relations rest. Participants often reported eating alone, separate even from their closest family. Altered eating therefore inevitably impacted on the patient’s sense of self and also identity. For some, the shared enjoyment of food and food-talk had been part of their social identity:

R2: I think for me it was absolutely massive [the impact of loss of ability to eat with other people], because I loved food, different flavours, different textures, wines…. people used to say that’s all I talked about.

Food, food culture, our sense of self and our social identity are closely linked [44]. Participant R2 was a self-confessed “foodie”; i.e., a person whose social activity and cultural identity were in part mediated by food. These aspects of identity was either gone, or had to be substantially adapted, in a way reminiscent of other “food minorities”:

R5: I always remember going out with vegetarians and you go round ten restaurants looking for something, and I’m like that now!

Specific culinary practices (being a vegetarian diet or a foodie, for example) position the eater within particular communities of practice. Altered eating, and therefore altered culinary practice, deprived patients of an important aspect of their social and cultural identity.

#### Emotion: The emotional life of the person is altered.

As a direct and cumulative impact of the foregoing anatomical, functional, sensory, cognitive, behavioural and social changes, there was a basic change in the emotional valence of food and in the emotional life of participants. By definition, food and eating are under the control of appetitive drives, i.e. have a valence of pleasure that motivates approach [45]. However, in this group of participants, this approach orientation and its driver of pleasure had been utterly altered. For some it was replaced by an acquired indifference, with concomitant loss of appetite:

R3: There’s no pleasure, so I’m starting to accept that that’s the way it’s going to be.

R3b: There’s very little flavour, so it’s not like there’s any incentive to try, because I don’t get any pleasure out of it….I’ve lost it altogether [appetite]. Yeah, I’m just not interested.

The behavioural and cognitive labour of eating in itself often deprives food of much of its pleasurable component. Food becomes mere nutrition, medicine to be taken in required but unpleasant doses. The link between emotion and food is also directly physiologically mediated. The olfactory system is linked to the amygdala-hippocampus complex; “the substrate of emotional memory” and memories evoked by odour are significantly more emotional than those recalled with visual cues [26,27]. Thus to have an altered relationship to food can result in a reduced ability to access pleasurable states, both past and present.

For participants who were still engaged with trying to eat, appetitive approach and pleasure had been replaced by “carefulness” and “caution” (R1). Food had become a source of danger (see functional section); a chore (see behavioural section); a problem to be solved (see cognitive section); an isolated anti-social experience (see social and cultural section). The overall emotional impact of these altered dimensions of food was considerable.

R1: It’s hard because you know what the stuff tastes like and it’s just been taken away from us.

R2: If it’s going down the wrong way, well maybe I will have to stop, but to say, I’m never going to eat again when I actually physically can eat a little bit, is, well, huge.

R2b: Mentally it has a big effect. It did with me because like I say I’ve seen a psychiatrist and all sorts, because I started going nuts, but you get over it, you get round it, you work with it. You’ve got to. What’s that saying, sink or swim?

A profound sense of loss of pleasure, frustration, sadness and distress were all in evidence. There was, within these groups, a collective mourning around the topic of food. Each participant had adopted a different stance to this. Some were still locked in a struggle with it:

R1: It’s extremely hard because I still refuse to accept it, because I still try food that I know it’s going to choke us.

Others had managed to compensate by finding their pleasures, commensality and social identity elsewhere:

R3: So, it’s sort of replaced what my social activities were of going out for meals with fitness and exercise, which is also good for you.

However, to end this section on a more hopeful note, almost all the participants remarked that the very act of coming together to discuss these issues and experiment with food with other survivors was in itself reparative, restoring some of the pleasurable, commensal aspects of food that had been lost. This finding also begins to indicate how this research might be useful in shaping interventions, a topic we will return to below.

## Discussion

Based on several years’ participant led work (including weekly small group discussions and many hours of detailed observation), two summative focus groups with participants, and respondent validation through public engagement events, we evolved a framework for addressing, assessing and, potentially, intervening in food related quality of life. An important part of this work was that it was participant led and extensively documented. Readers can see this framework “in action”, being discussed by the research participants in a short video film [28] (https://youtu.be/aMDI9bgRZ18); also see online supplementary material). Over the course of this work, it was clear that the impact of an alteration in eating extended well beyond the nutritional, and into areas of identity and social participation. We discerned seven key areas where the alteration of the relationship to food could manifest. Three of these were physiological: anatomical, functional and sensory. However, unlike much previous literature where the focus has been on the physical facticity of these elements, our research unearthed and emphasised qualitative research findings of their phenomenology in daily life: teeth that shatter as you eat, or disappear altogether following radiotherapy; salivary glands that are “fried” necessitating continual imbibing of food with water, a swallowing function that is not only altered by anatomy, but also by fear and the memory of choking in Frankie and Benny’s [a chain of Italian-American restaurants in the UK]. In the next two categories, we discovered how food could become cognitive and behavioural *hard labour:* a fact of everyday life that is often done on automatic, or with pleasure, had become instead a source of worry and effort; of planning and overly-prolonged execution. Food had become an ordeal, and one that required complete attention. The latter fact has particular ironic resonance in a health-care climate that suggests “mindful-eating” as an intervention to increase pleasure and decrease obesity. This group were forced into mindful eating. To let eating lapse into its habitual automatic register would be to entail the risk of aspiration, choking and possibly death. As such, the cumulative impact of this new labour intensive relationship to food was to profoundly alter the next category, social life and social identity. As much as participants mourned the loss of taste and flavour, they also mourned the loss of inclusion and belonging that food can afford. Finally, all the foregoing had a substantial emotional impact. Which was not to say that participants were depressed, but there was a universal sense that one area of pleasure, reward and comfort in life had been irrevocably altered. No-one was entirely reconciled to this loss of pleasure. Altered eating was, in this group at least, never just a matter of nutrition and its management. It was a phenomenon that profoundly affected many aspects of their lives, long after the disease and treatment that caused it were past.

Over the course of our work our research team (including the participants) came to define altered eating as: *a changed state of any combination of physical, emotional and social interactions with food and eating that has a negative impact on health and wellbeing.* It was clear that altered eating as a phenomenon was still a central feature of these cancer survivors’ lives yet, until the workshops, no-one in the health care system had addressed it. We would argue that this was because there was no systematic way of assessing and addressing it. We suggest that in

- identifying it as a phenomenon in its own right, and
- evolving a framework for its elucidation

we have developed a research and clinical concept that has potential to improve the lives of this and other patient groups.

It was clear from the group work and public engagement events that the framework might prove to be a useful framework for identifying relevant interventions for patients. For example, R3 (above) mentions taking up walking to replace what she missed about going out for meals with friends; it was clear that the framework provided a way of identifying compensatory strategies that could work for other patients. If anatomical, sensory and functional issues are intractable, the development of alternative sources of pleasure in the social/commensal area might help other patients adjust to their loss of pleasure and altered social participation and identity. As such, this framework could be used to clinically assess areas of deficit, and to identify areas of compensation and intervention.

As to identifying altered eating as a phenomenon, we believe that this is likely to be a trans-diagnostic entity. Work with other professionals and patient groups, from within our team, has already identified the potential usefulness of this framework in areas as diverse as ageing, Primary Sjogrens Syndrome, depression, weight cycling and non-head and neck cancer survivorship. Many people, as a result of disease or ageing, develop an altered relationship with food, and having a way of systematically diagnosing and intervening in this aspect of people’s lives may lead to improved quality of life in areas that are otherwise neglected. In current health care settings and research, food tends to show up under the category of nutrition. We believe that we have shown that its impact extends far beyond this, and that as researchers and health care professionals we need to fully address this impact.

## Conclusions

The Altered Eating Framework provides a systematic approach for assessing how the patient’s relationship to food has changed, the impact of this on their quality of life, and highlights areas for potential intervention. By highlighting the multi-factorial nature of our relationship to food, the framework also opens up the possibility for more creative interventions. This framework was iteratively co-produced with patients’ and the authorship of this paper includes a head and neck cancer survivor. We would prompt clinicians working with this and other conditions associated with altered eating to further test the framework’s utility as an assessment and treatment tool.

## Abbreviations

HNC: Head and neck cancer
PEG: Percutaneous endoscopic gastrostomy
PPI: Patient and public involvement
VRE: Video-reflexive ethnography
